# Promoter recognition specificity of *Corynebacterium glutamicum* stress response sigma factors σ^D^ and σ^H^ deciphered using computer modeling and point mutagenesis

**DOI:** 10.1007/s10822-024-00577-x

**Published:** 2024-11-25

**Authors:** J. Blumenstein, H. Dostálová, L. Rucká, V. Štěpánek, T. Busche, J. Kalinowski, M. Pátek, I. Barvík

**Affiliations:** 1 Institute of Microbiology, CAS, v.v.i, Prague, Czech Republic; 2 Center for Biotechnology (CeBiTec), Bielefeld University, Bielefeld, Germany; 3 Institute of Physics, Faculty of Mathematics and Physics, Charles University, Prague, Czech Republic

**Keywords:** Corynebacterium, Bio-orthogonal transcription, Promoter, Sigma factor

## Abstract

This study aimed to reveal interactions of the stress response sigma subunits (factors) σ^D^ and σ^H^ of RNA polymerase and promoters in Gram-positive bacterium *Corynebacterium glutamicum* by combining wet-lab obtained data and in silico modeling. Computer modeling-guided point mutagenesis of *C. glutamicum* σ^H^ subunit led to the creation of a panel of σ^H^ variants. Their ability to initiate transcription from naturally occurring hybrid σ^D^/σ^H^-dependent promoter P*cg0441* and two control canonical promoters (σ^D^-dependent P*rsdA* and σ^H^-dependent P*uvrD3*) was measured and interpreted using molecular dynamics simulations of homology models of all complexes. The results led us to design the artificial hybrid promoter P*D*_*35*_*H*_*10*_ combining the −10 element of the P*uvrD3* promoter and the −35 element of the P*rsdA* promoter. This artificial hybrid promoter P*D*_*35-rsdA*_*H*_*10-uvrD3*_ showed almost optimal properties needed for the bio-orthogonal transcription (not interfering with the native biological processes).

**Supplementary Information:**

The online version contains supplementary material available at 10.1007/s10822-024-00577-x.

## Introduction

*Corynebacterium glutamicum* is a non-sporulating gram-positive soil bacterium belonging to actinobacteria. Since its isolation in 1957 in Japan, *C. glutamicum* has been widely used as an industrial producer of amino acids and other useful substances such as diamines, carboxylic acids, polymers or biofuels [1, 2]. Strains of *C. glutamicum* also serve as expression systems for the production of heterologous proteins [3]. Due to their importance in biotechnology, production strains were bred both by classical and molecular methods. Regulation of transcription in *C. glutamicum* was studied in detail and its complete genome was finally sequenced [4].

Bacterial transcription is catalyzed by the RNA polymerase (RNAP) holoenzyme that consists of the multi-subunit core enzyme and an additional subunit called the sigma factor. Sigma factors are responsible for the recognition of promoters, local melting of dsDNA strands, and initiation of transcription [5].

*C. glutamicum* genome encodes 7 sigma factors (σ^A^, σ^B^, σ^C^, σ^D^, σ^E^, σ^H^, and σ^M^) [6]. All of them belong to the σ^70^-related family. Primary factor σ^A^, the only member of group 1 is responsible for transcription of housekeeping genes [6, 7]. Primary-like σ^B^ (group 2) is active mainly during the transition from exponential growth to stationary phase and in general stress response conditions [8]. Extra-cytoplasmic function (ECF) sigma factors σ^C^, σ^D^, σ^E^, σ^H^, and σ^M^ of group 4 are involved in various cell responses to stress [6].

The DNA sequence where RNAP binds and starts the transcription is called a promoter. Most of the bacterial promoters (housekeeping, vegetative) that are active during exponential growth are recognized by σ^A^ or less by σ^B^. They have two essential sequence elements (−35 and −10) that are mostly separated by the spacer with a length of 16–18 nucleotides. In most cases, the −10 element is AT-rich which allows the melting of the DNA double helix during the initiation of transcription [9–11]. The other promoters recognized by alternative σ factors have also two sequence elements (called generally −35 and −10 as well) that are variable, but highly conserved depending on the σ factor in control.

Different consensus nucleotide sequences of the −35 and −10 elements are thus recognized by various σ subunits with conserved amino acids that interact with particular nucleotides of the promoters. More specifically, the σ_4.2_ domain binds to the −35 element and the σ_2.4_ domain binds to the −10 element [12]. Despite substantial differences regarding the amino acid sequences of various σ subunits, the spatial arrangement of the σ_4.2_ and σ_2.4_ domains is highly conserved.

Although the number of crystal and cryo-EM structures of σ factors from various bacteria in the Protein Data Bank is steadily increasing, it has not yet been sufficiently elucidated which specific amino acids of σ subunits are crucial for the sequence-specific recognition of −10 and −35 promoter elements of various promoters in *C. glutamicum*.

We have long been interested in the functions of the stress subunits σ^H^ and σ^D^ of RNAP and the corresponding promoters in *C. glutamicum* (Busche et al., 2012; Taniguchi et al., 2017). Regarding σ^H^-dependent promoters, the GGAAT nucleotide sequence is typical in the −35 element and the consensus GTT sequence is usually found in the −10 element. The N_18–20_ spacer separates these elements. The analogous −35 and −10 elements of σ^D^-dependent promoters show the consensus sequence GTAAC-N_18–20_-GAT. In our previous study [13], we analyzed using computer modeling and point mutagenesis the naturally occurring hybrid σ^HD^-dependent *C. glutamicum* promoter P*cg0607*, having the GGAAC sequence (as a σ^H^-dependent promoter; σ^H^-matching) in the −35 element, and the GAT sequence (as a σ^D^-dependent promoter) in the −10 element. As expected, we found that the Lys-53-Ala mutation (transforming σ^H^ protein closer toward σ^D^) in the domain recognizing the σ^D^-like GAT in the −10 region of the P*cg0607* promoter resulted in substantially increased transcription with σ^D^. The 168-AlaValArgValAla-172 mutation in σ^H^ (i.e. again towards σ^D^) in the domain recognizing the σ^H^-matching −35 element GGAAC of the P*cg0607* promoter prevented transcription [13], in agreement with our assumption.

Here we followed up on our previous study [13] with the efforts to elucidate the contradictory points. We focused on another naturally occurring σ^D^/σ^H^-dependent hybrid promoter P*cg0441*, which has the CTAACG sequence close to the σ^D^ consensus at the −35 element and GTT that is σ^H^ consensus. We studied transcription from this promotor using a set of systematically mutated σ^H^ subunits designed based on computer modeling. Similarly, we analyzed in detail transcription from canonical σ^D^-dependent (P*rsdA*) and σ^H^-dependent (P*uvrD3*) promoters. Finally, we created an artificial hybrid promoter combining the −10 element from the P*uvrD3* promoter and the −35 element from the P*rsdA* promoter. This artificial hybrid promoter showed almost optimal properties in terms of bio-orthogonal transcription.

## Materials and methods

### Strains and cultivation conditions

The strain *Escherichia coli* TOP10 (Thermo Fisher Scientific Inc.) was used for cloning purposes. Wild-type strain *Corynebacterium glutamicum* ATCC 13032 was used as a host for the construction of two-plasmid clones containing pEPR1- and pEC-XT99A-derived recombinant plasmids. Plasmid PEPR1 [14] served as a promoter test vector and plasmid pEC-XT99A [15] served as an expression vector. *E*. *coli* was cultivated at 37 °C and *C. glutamicum* was cultivated at 30 °C, both in 2xYT medium or on 2xYT agar plates [16]

### DNA manipulations

PCR, plasmid DNA isolation, restriction enzyme digestion, ligation, and transformation of strains of *E. coli* were performed by using standard techniques [17]. Strains of *C. glutamicum* were transformed using electroporation [18]. In the case of the development of two-plasmid systems in *C. glutamicum*, the plates of 2 × YT medium were supplemented with tetracycline (5 µg/ml) and kanamycin (20 µg/ml) for selection of the transformants. DNA fragments for cloning promoters in pEPR1 were constructed of hybridized nucleotides with overhangs ready for ligation with vector digested by restriction enzymes BamHI and NsiI (New England Biolabs Inc.). Mutations in the *sigH* gene were performed by using the Q5 Site-Directed Mutagenesis Kit (New England Biolabs Inc.) according to the user guide of the manufacturer.

### Mutagenesis

All mutated *sigH* genes were produced using Q5® Site-Directed Mutagenesis Kit (New England BioLabs. Inc.) according to instructions of the manufacturer. PCR amplicons were produced with Q5 Hot Start High-Fidelity 2X Master Mix and appropriate oligonucleotide primers. The oligonucleotides for the mutagenesis were designed using the NEB online primer design software NEBaseChanger™ (NEBaseChanger.neb.com) and listed in supplementary Table 1S. The plasmids based on expression vector pEC-XT99A carrying the native *sigH* gene from *C. glutamicum* or its mutants were used as templates. Expression plasmids pEC-XT99A + x (x = *sigH*, *sigH*_4aa, *sigH*_6aa, σ^H^_4aa_K and σ^H^_6aa_K, respectively) were thus constructed.

### Promoter activity assay with two-plasmid* C. glutamicum* clones

To measure the promoter activity, a fluorescence intensity assay was used [19]. *C. glutamicum* culture inoculated in 2YT medium with antibiotics (30 µg/ml kanamycin, 10 µg/ml tetracycline) grown for 20 h at 30 °C was diluted into OD_600_ = 0.1. The cells were then cultivated on a shaker at 30 °C to OD_600_ = 1.0. After reaching OD_600_ = 1.0, approximately 15 ml of culture was taken from the flask to determine the "baseline specific fluorescence intensity" value. 30 ml of culture was then transferred to a new culture flask and supplemented with IPTG (time 0, fin. conc. 1 mM) for induction. The remaining culture was further cultured as a control of a non-induced sample.

Cells were harvested 3, 6, and 24 h after adding IPTG. Each harvest contained 7.2 mg of dry biomass. The sample was washed in PBS buffer supplemented with 1 mM phenylmethylsulfonyl fluoride (PMSF). All samples were homogenized by glass beads using FastPrep^Tm^ (MP Biomedical; 3 × 60 s at speed 6 m/s with Lysing Matrix B in 2-ml tubes). Homogenized samples were centrifuged and the supernatant was transferred into 96-well plate Nunclone™ Delta Surface and the fluorescence intensity was determined using a Saphire2 microplate spectrophotometer (Tecan; excitation wavelength 397 nm; emission wavelength 509 nm, excitation bandwidth 5 nm, emission bandwidth 20 nm, shake duration 5 s, settle time 10 s, read mode – bottom and integration time 40 µs). The protein concentration was determined using Bradford assays [20].

### Homology modeling and molecular dynamics simulations

The homology models of the σ^H^ and σ^D^ domains which recognize the −10 and −35 nucleotide sequences of the respective promoters were produced by using the Swiss-Model server [21]. The crystal structures of *E. coli* σ^E^, PDBid: 4LUP (for −10 element GTC) [22], and PDBid: 2H27 (for −35 element GGAAC) [23] were used as templates. The nucleotides within the consensus of the *E. coli* σ^E^-dependent promoters were replaced to match the consensus for *C. glutamicum* σ^H^- or σ^D^-dependent promoters, where necessary. Molecular dynamics (MD) simulations were done using the software package AMBER [24] and Linux computer nodes with powerful NVIDIA GPUs that enable the accumulation of 800-ns MD trajectories at 280 K.

## Results

### Choice of promoters and design of nucleotide alterations

In our previous study [13], we analyzed using computer modeling and point mutagenesis the naturally occurring H_35_D_10_-hybrid promoter P*cg0607*, having the σ^H^-matching sequence GGAAC in the −35 element and the σ^D^-matching GAT sequence in the −10 element (Table 1). The 168-AlaValArgValAla-172 mutation in σ^H^ in the domain recognizing the σ^H^-matching −35 element GGAAC of the P*cg0607* promoter (to make it more σ^D^-like) prevented transcription [13]. In other words, the naturally occurring H_35_D_10_ hybrid promoter P*cg0607* was not recognized by the artificially constructed hybrid σ^D^_35_^H^_10_ subunit.

**Table 1:** Nucleotide sequence of promoters used in this study

Gen	Nucleotide sequence	
*cg0607*	TTGCGCGTTAATAA**GGAAC**AATATCGGTGTGATTCGC**GAT**ATATTAATCA	H_35_D_10_
*cg0441* (*lpd*)	ATTTCGGCAGAGTG**CTAAC**GGTTAGGCACTATTTTCC**GTT**AGTTCTTTT**G**	D_35_H_10_
*uvrD3*	TTTAACCGCTATCT**GGAAT**GATTGATAGCTCCCAAGT**GTT**GTATCTATT**C**	σ^H^-dep
*rsdA*	GGTCAGCGATGGAA**GTAAC**AGAGTTAGGGAACTTCTC**GAT**CTACTGAGT**G**	σ^D^-dep
*rsdA_uvrD3*	GGTCAGCGATGGAA**GTAAC**AGAGTTAGGGTCCCAAGT**GTT**GTATCTATT**C**	D_35_H_10_

The conserved promoter −35 and −10 motifs are in bold underlined letters

These results led us to the idea that this σ^D^_35_^H^_10_ subunit could be used for transcription from artificial D_35_H_10_ hybrid promoters. In the best-case scenario, this hybrid promoter would be recognized neither by the σ^D^ nor σ^H^ native subunits and the transcription with the artificial σ^D^_35_^H^_10_ subunit would therefore be bio-orthogonal (not disturbing natural biological processes).

**Fig. 1:**
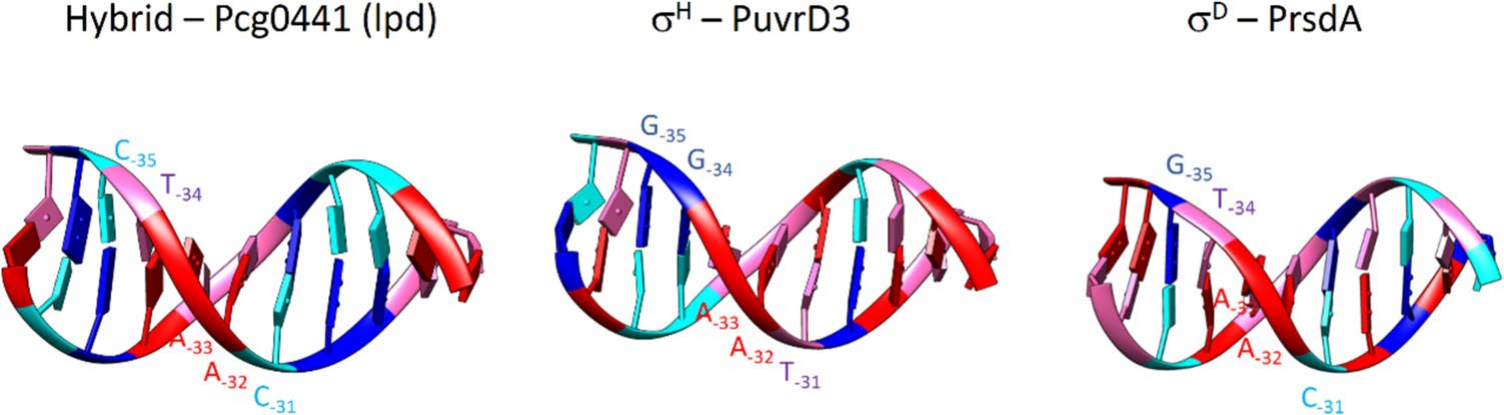
Duplexes consisting of 11 base pairs spanning positions −37 to −27 from the Pcg0441, PuvrD3, and PrsdA promoters. Color code: Adenine—red, Thymine—pink, Guanine—blue, Cytosine—cyan

First, we focused on the naturally occurring D_35_H_10_ hybrid promoter P*cg0441* (Table 1, Fig. 1), which has the CTAAC sequence close to the σ^D^-matching consensus (GTAAC) at the −35 element. Regarding the −10 element, the GTT sequence of P*cg0441* corresponds to the σ^H^-matching consensus promoter. Further, we analyzed in detail transcription from canonical σ^H^-dependent P*uvrD3* and σ^D^-dependent P*rsdA* promoters (Table 1, Fig. 1). Regarding σ^H^-dependent promoters, the GGAAT/N_18–20_/GTT sequence is nearly completely conserved in the −35/spacer/ −10 sequence (Fig. S1). The analogous −35 and −10 elements of σ^D^-dependent promoters show the consensus sequence GTAAC-N_17–18_-GAT.

Finally, we created an artificial D_35_H_10_ hybrid promoter combining the −35 element from the σ^D^-dependent P*rsdA* promoter and the −10 element from the σ^H^-dependent P*uvrD3* promoter (Table 1). Therefore, this promoter is hereinafter called the artificial D_35_H_10_ hybrid promoter P*D*_*35-rsdA*_*H*_*10-uvrD3*_.

### Design and creation of hybrid σ^D^_35_^H^_10_ from σ^H^ by point mutagenesis

**Fig. 2:**
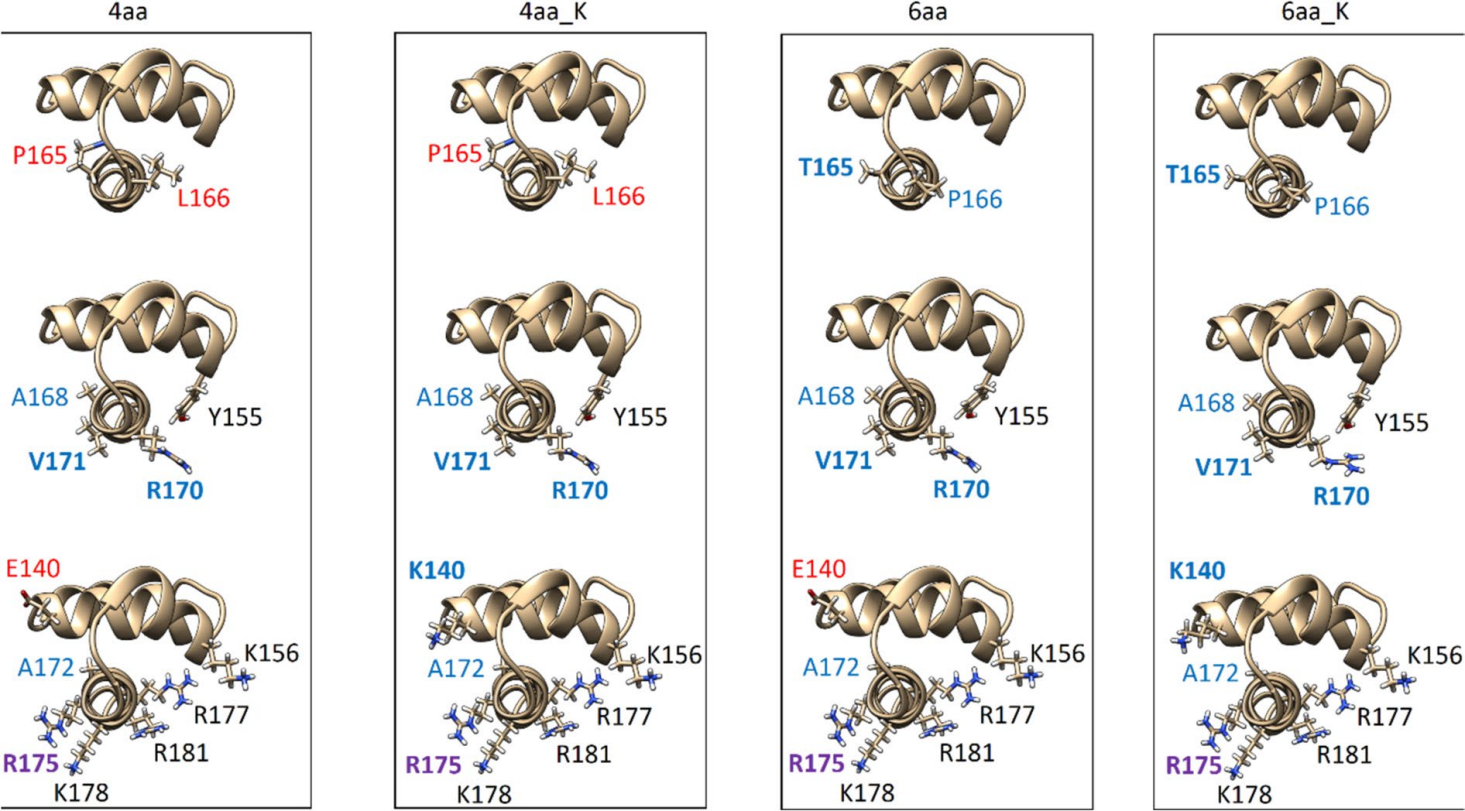
Domains 4.2 of mutant σ^H^ subunits interacting with the −35 elements of the studied promoters. σ^H^ amino acids have red labels, and mutated amino acids have blue labels. Labels of amino acids that appeared to be particularly significant in MD simulations are highlighted in bold. For clarity, the amino acids are shown separately: (*top*) those distinguishing the 4aa and 6aa mutants; (*middle*) amino acids from the initial 4aa mutation set that were mutated in all cases; (*bottom*) the amino acid at position 140, where K140 is supposed to compensate for the R172 to A172 mutation. Next, the crucial R175, which interacts with the nucleotide in position −34, and the basic amino acids K178, R171, R177, and K156, which are important for the interaction with the sugar-phosphate backbone of the nucleic acids

Transcription from the tested promoters (Table 1) was studied using a set of systematically mutated σ^H^ subunits (see Table 2, Fig. 2) designed on the base of computer modeling. These mutations are located in region 4 of σ^H^-based subunits which interacts with the −35 element. To avoid any confusion, amino acids through the following text are numbered according to *C. glutamicum* σ^H^ to highlight which amino acids are in spatially equivalent positions. For slightly shifted (2 aa) numbering of *C. glutamicum* σ^D^ subunit see Fig. 5 in [13], where sequence alignment with *E. coli* σ^E^ region 4 can be found as well (its structure PDB id: 2H27 served as a template for our homology models).

**Table 2:** Set of mutations in the region 4 of the *sigH* gene

Sigma factor	Name of mutation	Sequence change
H	4AA	T^168^M^170^S^171^R^172^ → A^168^R^170^V^171^A^172^
H	4AA + K	E^140^ T^168^M^170^S^171^R^172^ → K^140^ A^168^R^170^V^171^A^172^
H	6AA	P^165^L^166^T^168^M^170^S^171^R^172^ → T^165^P^166^A^168^R^170^V^171^A^172^
H	6AA + K	E^140^ P^165^L^166^T^168^M^170^S^171^R^172^ → K^140^ T^165^P^166^A^168^R^170^V^171^A^172^

Amino acids are numbered according to *C. glutamicum* σ^H^ to highlight which amino acids are in spatially equivalent positions

### Mutations

#### σ^H^_4aa

First, the σ^D^_35_^H^_10_ mutant from our previous study [13] was used. In this mutant, a set of amino acids 168-ThrValMetSerArg-172 in σ^H^ was replaced by 168-AlaValArgValAla-172 in the central helix of the σ^H^ domain which recognizes the −35 element [13]. Since V169 was not mutated, a total of just 4 amino acids were changed. Therefore, this mutant is hereinafter referred to as σ^H^_4aa. This variant was thought to be more σ^D^-like. Further mutations aimed to create an σ^D^_35_^H^_10_ mutant, which is even more σ^D^-like in the −35 binding region.

#### σ^H^_4aa_K

Our computer models based on P*cg0607* and the σ^H^_4aa mutant showed that the positively charged basic amino acid R172 in σ^H^, which was altered to provide σ^H^_4aa mutant, significantly interacted with the negatively charged phosphate groups in the sugar-phosphate backbone of the non-template-strand-of-DNA and formed thus salt bridges. Further, the model of a complex involving the σ^D^ subunit and P*cg0607* showed that another basic amino acid K140 in σ^D^ interacted with the identical phosphate groups as R172 in σ^H^. Interestingly, K140 of σ^D^ is situated outside the central helix, but its long and flexible side chain points to the same location as R172 in σ^H^.

Therefore, we decided to create the σ^D^_35_^H^_10_ variant, where in addition to the four amino acids mentioned above, the amino acid E140 was replaced by K140. This mutant is hereinafter referred to as σ^H^_4aa_K.

#### σ^H^_6aa

Two other amino acids in σ^H^ (T165, P166) and in σ^D^ (P165, L166) near the mutated σ^H^ sequence 168–172, are not conserved between σ^H^ and σ^D^. These amino acids undergo close contact with the non-template strand of DNA. Therefore, we created another σ^D^_35_^H^_10_ mutant, having a total of six amino acids altered. The amino acids 165-ProLeuGlyThrValMetSerArg-172 in σ^H^ were thus replaced by 165-ThrProGlyAlaValArgValAla-172 in this mutant named σ^H^_6aa. Note that Gly167 and Val169 are in both sigma subunits, and therefore, a total of just 6 amino acids were changed. Further, both hydroxyl groups of T168 and T165 aim for the same phosphate group of DNA. So the corresponding mutation at position 165 (Pro-165-Thr) was to ensure that the resulting mutant was not weakened compared to the parent σ^H^ when Thr168 was exchanged.

#### σ^H^_6aa_K

Finally, we merged all point mutations mentioned above in a construct hereinafter referred to as σ^H^_6aa_K.

To summarize, this study involves in total four promoters: P*cg0441*, P*uvrD3*, P*rsdA,* and P*D*_*35-rsdA*_*H*_*10-uvrD3*_ (Table 1), and six different σ subunits: σ^D^, σ^H^, σ^H^_4aa, σ^H^_4aa_K, σ^H^_6aa and σ^H^_6aa_K (Table 2).

### The activity of σ^D^ and σ^H^ and its mutant variants with promoters

In the biological part of this study, the ability of σ^D^, σ^H^, and all constructed σ^H^ mutants (σ^H^_4aa, σ^H^_4aa_K, σ^H^_6aa, and σ^H^_6aa_K - Table 2) to drive transcription (as subunits of RNAP) in all combinations with the four promoters (P*cg0441*, P*uvrD3*, P*rsdA,* and P*D*_*35-rsdA*_*H*_*10-uvrD3*_—Table 1) was tested by two-plasmid in vivo assay [19].

### (A) Natural D_35_H_10_ hybrid promoter P*cg0441*

**Fig. 3:**
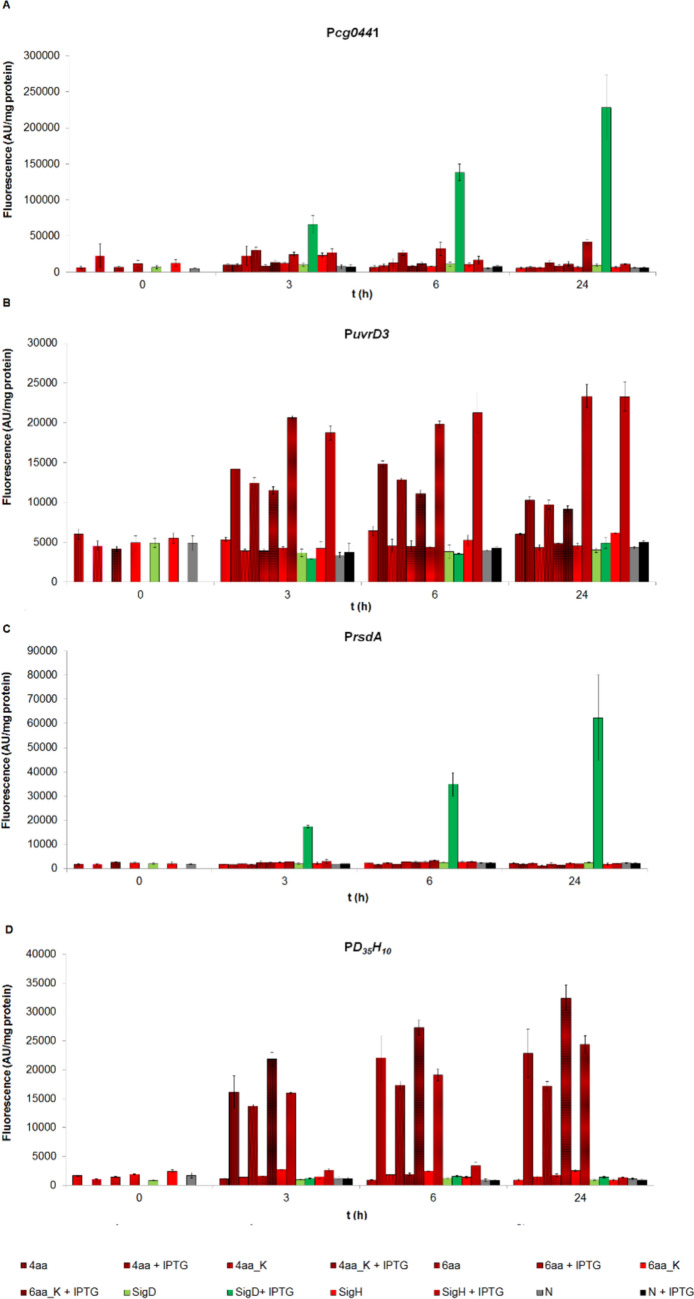
Determination of promoter activity in *C. glutamicum* WT with overexpressed σ^D^, σ^H^, and σ^H^ mutants (σ^H^_4aa, σ^H^_4aa_K, σ^H^_6aa, and σ^H^_6aa_K) by using the two-plasmid assay. The strain with the empty expression vector pECXT99A was used as a control (gray bars). The promoter activity was measured as the Gfpuv fluorescence intensity of the cell extracts. The fluorescence of cultures without IPTG is shown as light bars. The fluorescence of cultures with IPTG induction is shown as dark bars. AU, arbitrary units. The standard deviations of three biological replicates are depicted by error bars

While the activity of the natural D_35_H_10_ hybrid P*cg0441* promoter with the overproduced σ^D^ was high, it was almost undetectable with overproduced σ^H^ (Fig. 3a). The promoter activity with the σ^H^_4aa and σ^H^_6aa mutants was negligible as well. Nevertheless, it was slightly higher with the σ^H^_4aa_K and significantly with the σ^H^_6aa_K mutant. The natural D_35_H_10_ hybrid P*cg0441* promoter did not meet our requirements regarding its bio-orthogonality because it was strongly transcribed with natural σ^D^.

### (B) σ^H^-dependent promoter PuvrD3

In the case of the σ^H^-dependent P*uvrD3* promoter, all studied sigma subunits except σ^D^, initiated transcription (Fig. 3b). The σ^H^-dependent P*uvrD3* promoter activity was high with σ^H^ as expected. However, the non-negligible expression was observed more or less for all mutant σ^H^ subunits. It was not so much expected. In particular, it was surprising that a mutant of the σ subunit, the σ^H^_6aa_K, which is most similar to σ^D^, stimulated transcription strongly, even with the same intensity as the natural σ^H^ factor (Fig. 3b).

###  (C) σ^D^-dependent promoter P*rsdA*

Further, we examined transcription driven by the strong σ^D^-dependent P*rsdA* promoter (Fig. 3c). As expected, this promoter was active only in combination with σ^D^. For other σ factors, no increase in transcription above the basal level of fluorescence intensity was observed.

### (D) Artificial hybrid promoter P*D*_*35-rsdA*_*H*_*10-uvrD3*_

Promoter activity assay with the artificial/synthetic hybrid promoter P*D*_*35-rsdA*_*H*_*10-uvrD3*_ was high with all σ^H^ mutants (Fig. 3d). Equally important, however, was the finding that there was no increase in the P*D*_*35-rsdA*_*H*_*10-uvrD3*_ promoter activity in the presence of the overproduced σ^D^ factor (Fig. 3d). Similarly, there was almost no increase in the P*D*_*35-rsdA*_*H*_*10-uvrD3*_ promoter activity in the presence of overproduced factor σ^H^ (Fig. 3d). It means that both native σ^D^ and σ^H^ subunits did not recognize this artificial D_35_H_10_ hybrid promoter P*D*_*35-rsdA*_*H*_*10-uvrD3*_. Taken together, only engineered σ^H^ subunits with point mutations could initiate transcription. Thus, this artificial hybrid promoter P*D*_*35-rsdA*_*H*_*10-uvrD3*_ appears to have almost optimal properties needed for bio-orthogonal transcription.

Further, an equally important point is whether the mutant σ^H^ subunits induced any transcription from both native σ^D^- and σ^H^-dependent promoters (Fig. 3b and c). The requirement of missing activity was undoubtedly met for the σ^D^-dependent P*rsdA* promoter (Fig. 3c) since none of the mutant σ^H^ subunits showed any activity with P*rsdA* (Fig. 3c). In the case of the σ^H^-dependent promoter P*uvrD3*, the σ^H^_6aa mutant had the lowest and rather transient activity (Fig. 3b). It goes perfectly with the finding that the σ^H^_6aa mutant has the highest activity with the artificial/synthetic hybrid promoter P*D*_*35-rsdA*_*H*_*10-uvrD3*_.

The σ^H^_6aa subunit and artificial/synthetic hybrid promoter P*D*_*35-rsdA*_*H*_*10-uvrD3*_ thus seem to be the most suitable candidates for creating the σ-promoter pair, which can ensure bio-orthogonal transcription.

To sum up, the wet-lab experiments demonstrated that we achieved the main goal, to construct the bio-orthogonal transcription. However, many partial results were in various aspects rather unexpected and counter-intuitive, especially in the case of the natural hybrid P*cg0441* promoter and the σ^H^-dependent P*uvrD3* promoter. Therefore, in the following paragraphs, we will try to decipher and interpret these partial results using homology modeling and MD simulations. We consider the compliance of the results of the in vivo measurements with the computational models the crucial point of the study that provides clues for future development and fine-tuning of bio-orthogonal D_35_H_10_ hybrid σ subunits and promoters.

## Computer modeling

In the following paragraphs, we summarize the findings on the crucial nucleotides and amino acids involved in mutual interactions that were achieved using our computer models. Further, we present here the results of extensive MD simulations of 18 complexes covering all possible combinations of six σ_4_ segments (see Table 2), and three −35 elements derived from our four promoters (σ^D^-dependent P*rsdA* and synthetic hybrid promoter P*D*_*35-rsdA*_*H*_*10-uvrD3*_ share the same −35 element—see Table 1). Finally, we will provide tentative explanations of the observed biological activities (see Fig. 3).

**Fig. 4:**
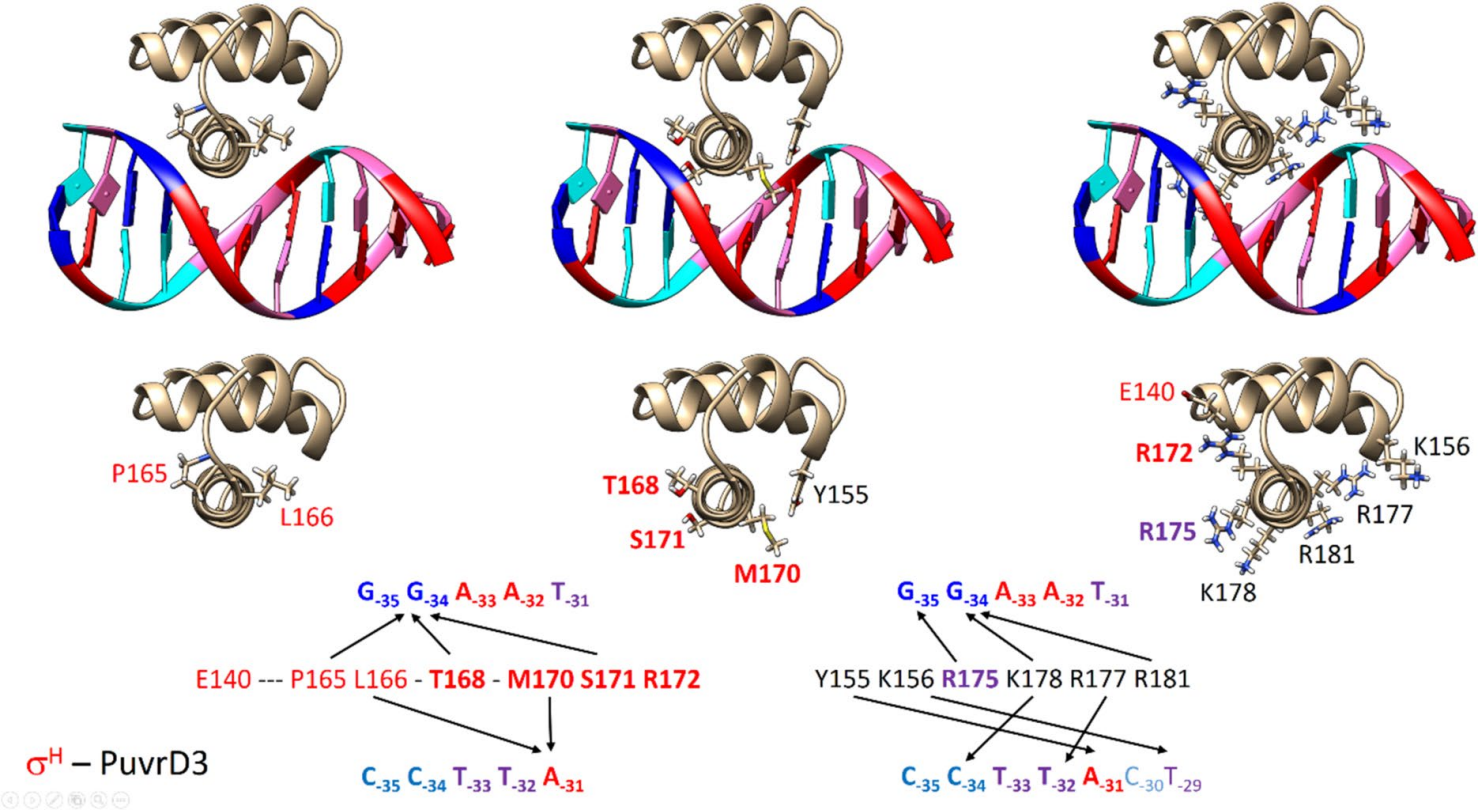
Domain 4.2 of the σ^H^ subunit interacting with the −35 element of the PuvrD3 promoter. Amino acids from σ^H^ that were mutated in this study have red labels. Labels of amino acids that significantly interacted with the promoter in MD simulations are highlighted in bold. For clarity, amino acids are shown separately: (*left*) mutated only in 6aa mutants; (*middle*) mutated in all cases; (*right*) amino acid E140, which was mutated in the 4aa_K and 6aa_K mutants to compensate for the R172 to A172 mutation. I.e. so that a positively charged amino acid side chain is present at the site that could interact with the negatively charged backbone of the nucleic acid. In addition, R175, which interacts with the nucleotide base at position −35, as well as the basic amino acids K178, R171, R177, and K156, which are important for interaction with the sugar-phosphate backbone of the nucleic acids, are shown here

**Fig. 5:**
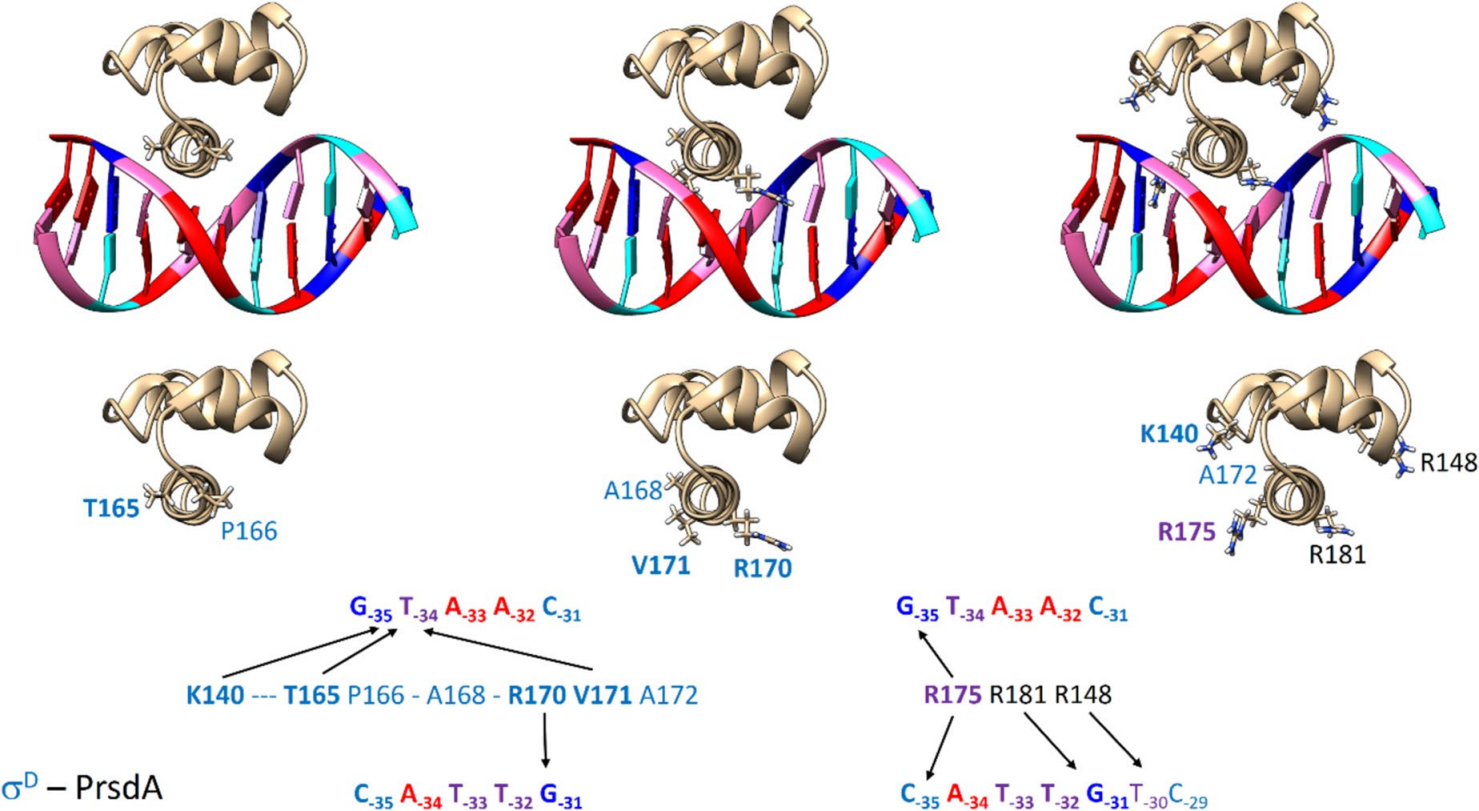
Domain 4.2 of the σ^D^ subunit interacting with the −35 element of the PrsdA promoter. Amino acids from σ^D^ that were mutated have blue labels. Labels of amino acids that appeared to be particularly important in MD simulations are highlighted in bold. For clarity, amino acids are shown separately: (*left*) mutated only in 6aa mutants; (*middle*) mutated in all cases; (*right*) K140 from the 4aa_K and 6aa_K mutants that compensates for the R172 to A172 mutation. Also shown is the crucial R175 interacting with the nucleotide base at position −35 and the basic amino acids R181 and R148, which are important for interaction with the sugar phosphate backbone of the nucleic acids

Our computer models (Figs. 4, 5) show that interactions between amino acids and nucleotides can be divided into sequence-specific, which involve nucleobases, and sequence non-specific, which involve negatively charged sugar-phosphate backbone of DNA attracting long and flexible side-chains of basic amino acids (arginines or lysines). Further, amino acids with hydroxyl groups that can form hydrogen bonds (for example Y155 or T165) are also involved.

### Nucleotides involved in σ_4_ region–promoter interactions

In the promoters, P*cg0441,* P*uvrD3,* P*rsdA*/P*D*_*35-rsdA*_*H*_*10-uvrD3*_, the non-conserved bases at the first and second (i.e. −35, −34) positions within the −35 elements of the non-template DNA strand (**CT**AAC/**GG**AAT/**GT**AAC)and the non-conserved base at the fifth (i.e. −31) position of the opposite (template) DNA strand (GATT**G**/CCTT**A**/CATT**G**) were found to participate in the most important sequence-specific interactions with polar amino acids of σ_4_ subunits (Figs. 4, 5).

Specifically, the C_−35nt_T_−34nt_–G_−31t_ bases within the σ^D^_35_H_10_-dependent promoter P*cg0441*, are involved in these contacts. In the case of the σ^H^-dependent promoter P*uvrD3*, the G_−35nt_G_−34nt_-A_−31t_ bases are involved. Finally, in the case of the σ^D^-dependent P*rsdA*, and the hybrid promoter P*D*_*35-rsdA*_*H*_*10-uvrD3*_, the G_−35nt_T_−34nt_–G_−31t_ bases are involved (Figs. 4, 5).

Furthermore, the conserved bases −33, −32 (more specifically AA/TT in the non-template/template DNA chain) are in close contact with the σ_4.2_^H^/σ_4.2_^D^ helix between the mutated amino acids at positions 170 and 171 (i.e. M170, S171 or R170, V171). The methyl groups of both T bases participate in stabilizing hydrophobic interactions, especially with the bulky non-polar side-chain of V171.

Moreover, several nucleotides surrounding the canonical −35 element may be in at least occasional contact with mutated amino acids. This may to some extent modulate the recognition between specific σ-subunits and promoters. For example, the −30 nucleotide interacts with the amino acid R170 or M170, or the −37, and −36 nucleotides interact with the amino acid K140.

### Amino acids involved in σ_4_–promoter interactions

The central helix of the σ_4.2_^H^/σ_4.2_^D^ segments is involved in interactions with the −35 elements of promoters.

The G/A base at position **−31** of the template strand of DNA interacts with R170 at σ^D^. In particular, if G is in position −31, up to two stabilizing hydrogen bonds may be formed. Alternatively, the long flexible side chain of R170 can create the so-called salt bridge with negatively charged phosphate groups of the sugar–phosphate backbone of DNA.

In the case of σ^H^, the G/A base at position **−31** interacts with M170. Methionine cannot form hydrogen bonds with bases or salt bridges with phosphate groups of the sugar–phosphate backbone of DNA like R170. However, the M170 side chain forms a tight cluster with the side chains of amino acids L166, Y155, and R177 (not present in σ^D^). The latter two amino acids interact with the sugar–phosphate backbone of DNA. This seems to be how the presence of an either optimal or suboptimal interaction partner i.e. A_−31_ or G_−31_ in the vicinity of M170 can result in either stabilization or destabilization of the σ–promoter complex.

**Fig. 6:**
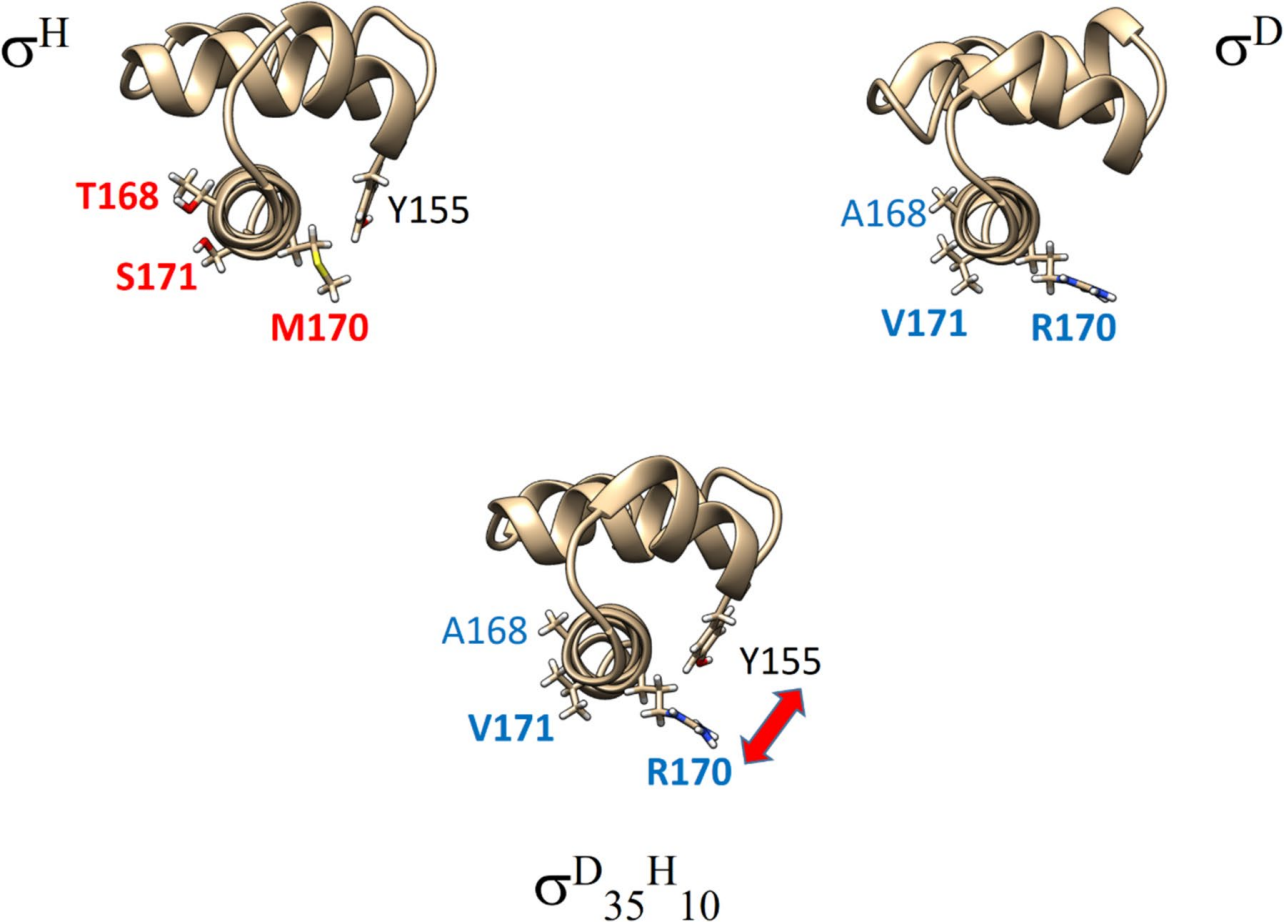
In the case of mutant σ^D^_35_^H^_10_ subunits (bottom), a specific situation occurs. There is R170 transferred from σ^D^ (top right) but there is also amino acid Y155 from σ^H^ (top left). In MD simulations, it took quite a while before the long side chains of R170 and Y155 relaxed and settled properly into an arrangement without steric clashes between them. More specifically, the optimal interaction of R170 with base −31 was mostly observed when spatially close Y155 was not bound to the phosphate group of DNA. Steric conflicts between R170 and Y155 could be one of the reasons, why we generally observed weaker transcription with the σ^D^_35_^H^_10_ mutants than was achieved with σ^D^ that lacks Y155

In the case of mutant σ^D^_35_^H^_10_ subunits, a specific situation occurs (Fig. 6). There is R170 transferred from σ^D^, but there are also amino acids L166, Y155, and R177 from σ^H^. In our MD simulations, it took usually quite a while before long side chains of R170 and Y155 relaxed and settled properly into an arrangement without steric conflicts/clashes between them.

**Fig. 7:**
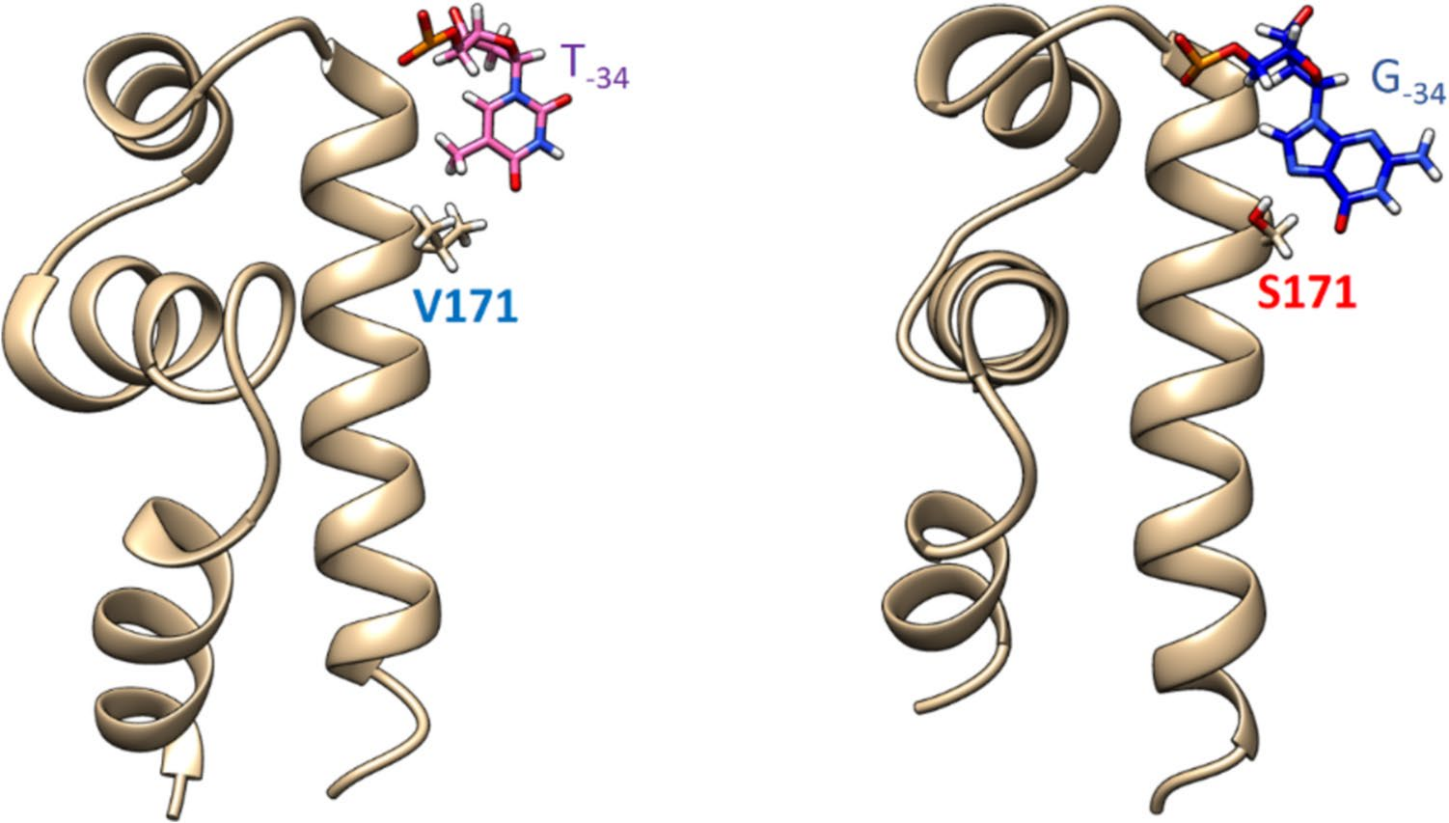
The G/T bases at position** −34** of the non-template DNA strand interact with amino acids that are specific for σ^H^ (S171] and σ^D^ (V171) subunits. While S171 forms stabilizing hydrogen bonds with the G_−34_ base, the attraction of V171 and T_−34_ stems from their hydrophobicity

The G/T bases at position −**34** of the non-template DNA strand interact with amino acids that are specific for σ^H^ (S171) and σ^D^ (V171) subunits (Fig. 7). While S171 forms stabilizing hydrogen bonds with the G_–34_ base, the attraction of V171 and T_–34_ stems from their hydrophobicity.

Close contacts of atypical interacting partners S171 and T_−34_ (in σ^H^ complexes with the σ^D^-dependent promoter P*rsdA*, natural D_35_H_10_ hybrid promoter P*cg0441,* and artificial hybrid promoter P*D*_*35-rsdA*_*H*_*10-uvrD3*_) are destabilizing. This is because a hydrogen bond formed between the hydroxyl group of S171 and oxygen atom O4 of T_–34_ leads ultimately to the disruption of other stabilizing interactions.

In contrast, close contacts of atypical interacting partners V171 and G_–34_ in complexes of σ^**D**^ and σ^D^_35_^H^_10_ mutants (σ^H^_4aa, σ^H^_4aa_K, σ^H^_6aa, and σ^**H**^**_6aa_K**) with the σ^H^-dependent promoter P*uvrD3*, don't seem to be destabilizing.

**Fig. 8:**
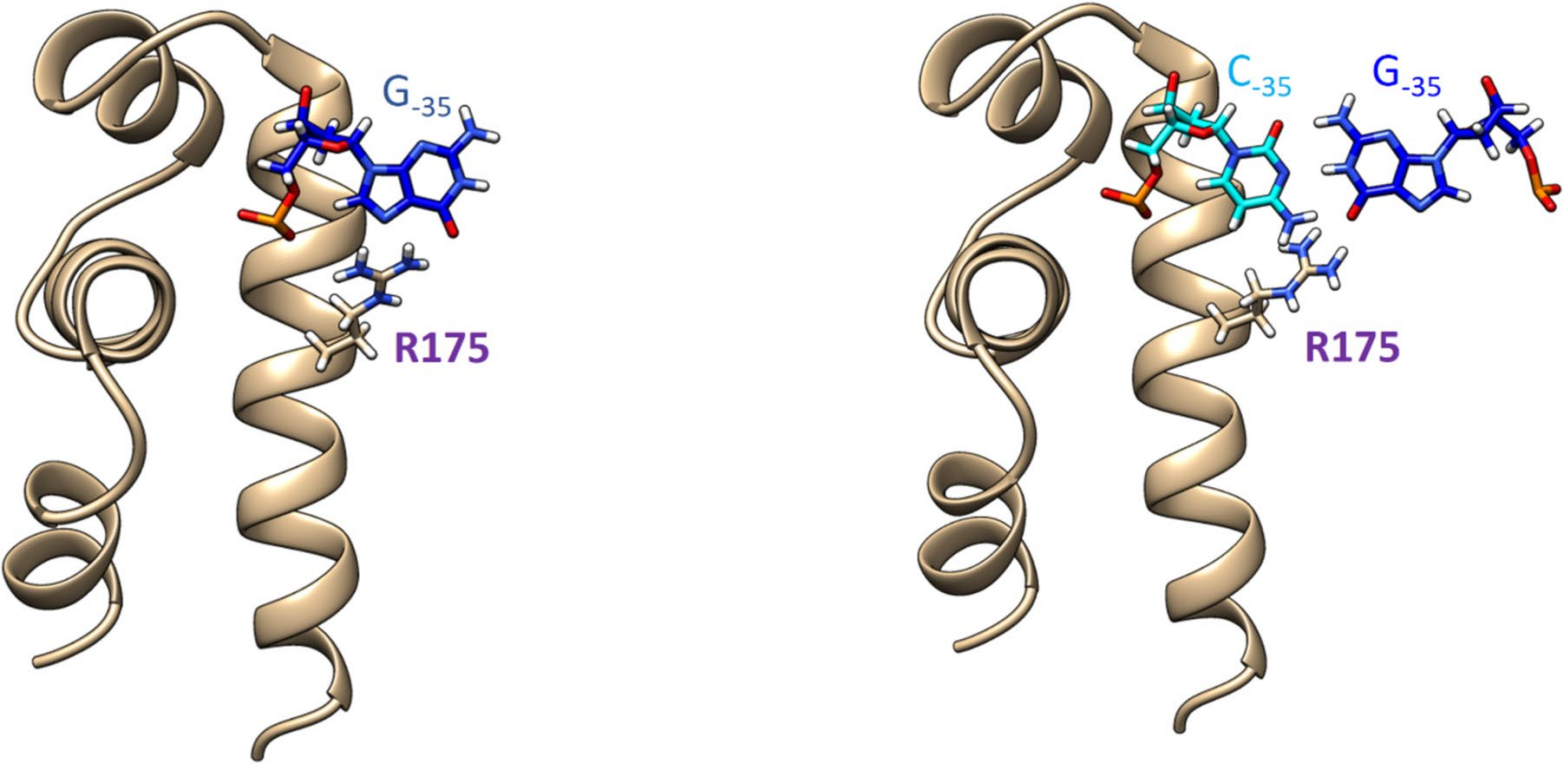
(Left) Base G at the position −**35** of the non-template DNA strand of either σ^H^-dependent P*uvrD3* or σ^D^-dependent P*rsdA* interacts (via two hydrogen bonds involving atoms O6 and N7) with an arginine side chain R175, which is present in the equivalent spatial position of both σ^H^ and σ^D^ subunits. (Right) The C base at the first position of the Pcg0441 −35 element does not allow usual hydrogen bonding with the R175 side chain. Instead, this R175 side chain interacts with the guanine base at position −35 in the complementary template strand of DNA

Base G at the position −**35** of the non-template DNA strand of either σ^H^-dependent P*uvrD3* or σ^D^-dependent P*rsdA* interacts (via two hydrogen bonds involving atoms O6 and N7) with an arginine side chain R175 (Fig. 8), which is present in the equivalent spatial position of both σ^H^ and σ^D^ subunits. These hydrogen bonds with the involvement of R175 (conserved in σ^H^ and σ^D^) we consider to be the most important. After their disruption, a substantial repositioning of the DNA:DNA duplex relative to σ_4_ domains usually followed quickly.

As regards sequence non-specific interactions, R181 of both σ_4_^H^/σ^D^ domains interact with the negatively charged sugar–phosphate backbone of DNA chains and provide further stabilization of σ_4_–promotor complexes. Further, there is another basic amino acid K178, which is present only in σ_4_^H^ and in its mutants.

### Stability of σ_4_^D^–promoter complexes in MD simulations

Let us summarize the results of 800 ns MD simulations for 18 simulated systems grouped based on the involved σ_4_ subunits.

### Interactions of σ_4_^H^ with promoters

As expected, σ_4_^H^ created the most stable complex with the σ^H^-dependent promoter P*uvrD3*. All possible stabilizing interactions mentioned above were found: sequence-specific (G_–35_–R175, G_–34_–S171, A_−31_–M170) as well as sequence non-specific (i.e. mediated by binding of side chains of Y155, R177, K156, R172, T168, R181, K178 to the sugar–phosphate backbone of nucleic acids).

On the other hand, in σ^D^- and σ^D^/σ^H^-dependent promoters (i.e. P*rsdA*, P*cg0441,* and P*D*_*35-rsdA*_*H*_*10-uvrD3*_), most of these interactions were interrupted, the orientation of the DNA:DNA duplex relative to σ_4_^H^ was significantly changed, or even this complex was completely abrogated. In any case, hydrogen bonding between undue partners (S171 and T_–34_) was a primary destabilizing factor.

### Interactions of σ_4_^D^ with promoters

All complexes were quite stable and only small deviations were observed for σ_4_^D^, expected stabilizing interactions mentioned above were observed: sequence-specific (C/G_–35_–R175, T_–34_–V171, G_–31_–R170) as well as sequence non-specific (i.e. mediated by binding of side chains of R148, E156, K140, T165, R181 to the sugar phosphate backbone of nucleic acids).

The only exception was the σ^H^-dependent promoter P*uvrD3*, where significantly greater disturbance of interactions was transiently observed, and it is questionable whether this complex would remain stable during a longer MD simulation.

### Interactions of σ_4_^H^ mutants with promoters

Mutations of only 4–7 amino acids that bring σ^H^ closer towards σ^D^ (resulting in σ^H^_4aa, σ^H^_6aa, σ^H^_4aa_K, or σ^H^_6aa_K constructs) were sufficient for the stabilization of complexes of all mutated σ^H^ subunits with σ^D^- and σ^D^/σ^H^-dependent promoters (P*rsdA*, P*cg0441* and P*D*_*35-rsdA*_*H*_*10-uvrD3*_).

On the contrary, due to these mutations, certain destabilization of complexes with the σ^H^-dependent P*uvrD3* promoter occurred. Here, the most stable was the complex with σ^H^_6aa_K,

The optimal interaction of R170 with base -31 was mostly observed when spatially close Y155 was not bound to the phosphate group of DNA. Steric conflicts between those two amino acids could be one of the reasons, why we generally observed weaker transcription with the σ^D^_35_^H^_10_ mutants than was achieved with σ^D^ that lacks Y155.

## Interpretation of experimental results based on homology models and MD simulations

### (A) Natural PD_35_H_10_ hybrid promoter Pcg0441

While the activity of the P*cg0441* promoter with the overproduced σ^D^ was high, it was almost undetectable with overproduced σ^H^ in the biological assays (Fig. 3A). In MD simulations, the complex of the −35 element of P*cg0441* with σ_4_^D^ was stable, while the complex with σ_4_^H^ was unstable—apparently due to the stabilizing interaction of the T_–34_ base with V171 (σ_4_^D^) or destabilizing interactions of the T_–34_ base with S171 (σ_4_^H^).

Although experimental results and MD simulations are in agreement, the fact that P*cg0441* is strongly σ^D^-dependent is not entirely obvious given that the GTT sequence in the −10 element is typical for σ^H^-dependent promoters. Nevertheless, the completely inappropriate −10 element of P*cg0441* was not an obstacle for σ^D^ to very strongly stimulate transcription from this natural hybrid promoter.

A detailed analysis of MD trajectories showed that this could be related to the atypical base at the first position of the −35 element (in the non-template strand of DNA). Here, the P*cg0441* promoter has the C base. It means that it carries sequence CTAAC instead of GTAAC canonical for σ^D^-dependent promoters.

Our MD simulations showed that the C base at the first position of the P*cg0441* −35 element does not allow usual hydrogen bonding with the R175 side chain. Instead, this R175 side chain interacts with the guanine base at position −35 in the complementary template strand of DNA. In addition, the R175 side chain also interacts with the guanine base in the non-template DNA strand, however, at position –36 (i.e., at the position immediately preceding the −35 element).

This atypical interaction of R175 has effects on the interactions of R170. The R170 side chain ideally contributes to sequence-specific recognition of the base at the fifth position of the −35 element (specifically in the template strand). Here, however, the R170 side chain prefers sequence-non-specific interactions with phosphate groups in the sugar–phosphate backbone of the DNA template strand. In other words, a salt bridge is formed.

R170 is the last amino acid that substantially interacts with the −35 element. In structures of open RNAPs with resolved complete transcription bubbles, a ~ 17–18 bp spacer DNA:DNA duplex connecting the −35 and −10 elements follows (Table 2). It seems that subtle rearrangements of key interactions between P*cg0441* and σ subunits that appear due to the atypical C base at the first position of the −35 element are somehow transmitted through R170 and the spacer duplex toward the −10 element region, where it could slightly disturb spatial RNAP–DNA arrangement as well as underlying interactions between amino acids and nucleotides. Among other things, this could cause σ^D^ to be able to recognize GTT (typical rather for σ^H^-dependent promoters) in the −10 element of the natural PD_35_H_10_ hybrid promoter P*cg0441*.

Further, the natural PD_35_H_10_ hybrid promoter P*cg0441* activity with the σ^H^_4aa and σ^H^_6aa mutants was negligible. Transcription was slightly higher with the σ^H^_4aa_K and especially with the σ^H^_6aa_K mutant. However, it fell far short of the level of transcription observed with σ^D^. We attribute this mainly to the fact that in MD simulations the crucial R170 was not able to establish an interaction with the base G_–31_ or with a nearby phosphate group (as was the case with σ^D^). The obvious reason was the side chain of Y155 (which is missing in σ^D^, where it is replaced by the less bulky A155), with which R170 is in very close contact that disrupts to some extent interactions of both Y155 and R170 with nucleic acids.

Overall, the natural *PD*_*35*_*H*_*10*_ hybrid promoter P*cg0441* was a bit of a disappointment to us, as it did not meet our expectations as regards the level and bio-orthogonality of transcription achieved with mutant σ subunits. However, the atypicality of P*cg0441* concerning its base at position −35 and the resulting differences in the arrangement of the σ_4_-promoter complexes found in the MD simulations raised our hope that a hybrid *PD*_*35*_*H*_*10*_ promoter is in principle possible to create.

### (B) σ^H^-dependent promoter PuvrD3

In the case of the σ^H^-dependent P*uvrD3* promoter, all studied sigma subunits except σ^D^ more or less initiated transcription (Fig. 3b). Nevertheless, natural σ^H^ and σ_4_^H^_6aa_K stimulated transcription by far the most.

Our MD simulations show that not only natural σ_4_^H^ and σ_4_^H^_6aa_K but also σ_4_^D^can recognize the −35 element of the P*uvrD3* promoter. Nevertheless, in the case of σ_4_^D^, the crucial interaction between the R175 side chain and the base at the −35 position was remarkably less stable. R170 from ^D^ was not able to establish stable contact with the phosphate group of the DNA backbone, as was the case with σ_4_^H^_6aa_K. MD simulation showed that this was again partly due to the absence or presence of Y155. Nevertheless, Y155 now had opposite effects on R170 than in the case of σ_4_^D^ vs. σ_4_^H^_6aa_K and the P*cg0441* promoter.

### (C) σ^D^-dependent promoter PrsdA

The strong σ^D^-dependent P*rsdA* promoter was active only in combination with σ^D^ (Fig. 3c). For other σ factors, no increase in transcription above the basal level of fluorescence intensity was observed.

Our MD simulations showed that except σ_4_^H^ all other studied σ_4_ subunits can recognize the −35 element of the σ^D^-dependent P*rsdA* promoter. The crucial interaction between the R175 side chain and the base at the −35 position of the −35 element was stable leading to the classic arrangement of complexes.

The *in-vivo* inability of mutant σ^D^_35_^H^_10_ subunits to stimulate transcription from this σ^D^ promotor probably lies in their inability to recognize the GAT nucleotide sequence in the −10 element, which is typical for σ^D^-dependent promoters.

### (D) Artificial hybrid promoter PD_35-rsdA_H_10-uvrD3_

Finally, we used the P*uvrD3* and P*rsdA* promoters to construct an artificial hybrid P*D*_*35-rsdA*_*H*_*10-uvrD3*_ promoter that combines the canonical −35 element of the σ^D^-dependent P*rsdA* and the canonical −10 element of the σ^H^-dependent P*uvrD3*. Promoter activity assay with this synthetic hybrid promoter P*D*_*35-rsdA*_*H*_*10-uvrD3*_ provided high transcriptional activity with all σ^H^ mutants (Fig. 3d).

This confirmed our hypotheses that just the incompatible −10 element of P*rsdA* (Fig. 3c) and the atypical −35 element of natural PD_35_H_10_ hybrid promoter Pcg0441 (Fig. 3a) hindered transcription of these promotors using our mutant σ^H^ subunits.

Both native σ^D^ and σ^H^ subunits did not recognize the hybrid P*D*_*35-rsdA*_*H*_*10-uvrD3*_ promoter (Fig. 3d). Thus, this artificial promoter appears to have optimal properties needed for bio-orthogonal transcription.

None of the mutant σ^H^ subunits show any activity with σ^D^-dependent P*rsdA*. Further, in the case of the σ^H^-dependent promoter P*uvrD3*, the σ^H^_6aa mutant had the lowest activity (Fig. 3b).

Interestingly, this σ^H^_6aa mutant has the highest activity with the artificial hybrid promoter P*D*_*35-rsdA*_*H*_*10-uvrD3*_.

Taken together, the σ^H^_6aa subunit and P*D*_*35-rsdA*_*H*_*10-uvrD3*_ promoter seem to be the most suitable candidates as a σ-promoter pair that could enable bio-orthogonal transcription.

Prospectively, another designed mutation in the σ^H^_6aa subunit, i.e., Y155A, could decrease the unwanted transcription from the σ^H^ promoter P*uvrD3* and increase the transcription driven by the P*D*_*35-rsdA*_*H*_*10-uvrD3*_ hybrid promoter to the level that was observed with σ^D^ and the σ^D^-dependent promoter P*rsdA*.

## Discussion and conclusions

*C. glutamicum* has been applied in various biotechnological processes for the production of various amino acids, value-added chemicals, fuels, and polymers [3, 25]. To make these processes more effective, the production/transcription by bacteria should take place bio-orthogonally, to interfere as little as possible with their metabolism. For this, it is necessary to create pairs of artificial promoters and sigmas (or also anti-sigmas). This can be achieved in several different ways. Orthogonal pairs can be found in the genomes of various bacteria. Moreover, such regulatory units can be created artificially as chimeras or more subtly through amino acid point mutagenesis.

For example, 86 ECF σs, their promoters, and 62 anti σs identified from the genomes of diverse bacteria were used to identify a subset of 20 σs and promoters that were found to be highly orthogonal to each other [26]. They were used to build synthetic genetic switches in *Escherichia coli* [26]. Further, it was proposed by Rhodius et al. that this set could be extended by combining the −35 and −10 binding domains to build chimeras.

Indeed, a synthetic hybrid σ combining the −10 DNA binding domain of σ^70^ with the −35 DNA binding domain of σ^32^ was able to recognize a cognate hybrid promoter containing a consensus σ^32^ −35 motif and a consensus σ^70^ −10 motif [27].

Recently, 91,552 potential promoters belonging to 41,665 unique ECF σs were studied in detail [28]. Motifs were aligned, and putative promoter sequences were associated with the respective ECF σ sequences. Only a few amino-acid + nucleotide pairs were identified as important, and these were consistent with specificity-determining interactions observed in the crystal structures of three divergent ECF σs bound to their cognate promoters [29–31]. It suggests that the mechanism of ECF σ-promoter interaction is conserved in a majority of ECF σs despite their extensive sequence divergence. Artificial σ subunits can be therefore tailored to be specific for the respective hybrid promoters through point mutations of a very small number of amino acids.

Here we followed up on our previous study [13] and first looked at naturally occurring hybrid promoter P*cg0441*, which has a sequence CTAACG close to the consensus of the σ^D^-dependent promoters in the −35 region and GTT in the −10 region which conforms the σ^H^-dependent promoters. We studied transcription driven from this promotor using a set of systematically mutated σ subunits based on σ^H^. The mutations were proposed purely based on homology models constructed using the few available crystal structures of ECF σs, but it is clear that these mutations fall within a region recently identified as crucial in the study of tens of thousands of promoters and ECF sigma factors mentioned above see Fig. 1b in [28].

The most extensively mutated σ^H^ subunit, σ^H^_6aa_K, was the most potent one from this set as regards transcription from the natural hybrid P*cg0441* promoter. This finding confirmed our assumption that all proposed mutations are meaningful and bring the expected and synergistic effect. In other words, it means that σ^H^ with a few point mutations is undoubtedly able to interact effectively with the −35 element carrying the nucleotide sequence typical for σ^D^-dependent promoters.

On the other hand, our experimental results clearly showed that the naturally occurring hybrid promoter P*cg0441* is by no means suitable for bio-orthogonal transcription, as it is strongly recognized by the native σ^D^ factor (despite the GTT sequence in the −10 region which is typical for σ^H^-dependent promoters and which should prevent σ^D^ binding).

We believe that σ^D^ can recognize this naturally occurring hybrid promoter P*cg0441* partly due to the atypical base sequence in the −35 element region, specifically due to the base C_−35_ at the first position of the −35 element consensus sequence (**C**TAAC). MD clearly showed that this leads to a remarkably distorted binding of this atypical −35 element to σ subunits. We suppose, that this could ultimately affect the conformation of the whole spacer region of upstream DNA and even the binding of the distant −10 element of upstream DNA to the σ_2_ segment of sigma subunits.

Indeed, although there are only a few structures of the entire RNAP in complex with the ECF σ subunit and promoter DNA, they show the remarkable conformational variability of the ECF σ subunits in the region recognizing the −35 element (compare PDBid: 6JBQ, 6CA0, 5ZX2 – [29–31]). Different ECF σ subunits use a distinct hydrophobic surface to bind the tip helix of RNAP-β flap domain that rotates by 90°. This is accompanied by different bending of the DNA in the spacer region and by changes in its interaction with the positively charged amino acids of RNAP. Some distortions are visible even in the parts of the complexes, where σs recognize the −10 element. Thanks to the flexibility of the σ_4_ segments of the sigma subunits, transcription can probably start from promoters with spacers of different lengths or with certain deviations from consensus sequences in the −35 element. Nevertheless, it should be taken into account that these observations are based on a comparison of structures gained by different experimental techniques, namely cryoEM (6JBQ) and X-ray (5ZX2). Therefore, more structures obtained through cryo-EM will have to be awaited before making conclusions. Alternatively, extensive MD simulations (on a time scale of at least us), during which the 5ZX2 structure would be allowed to relax, could clarify whether the spacer DNA and tip helix of RNAP-β flap domain undergoes conformational changes exclusively due to the contacts with different sigma subunits or if it is partly a consequence of the so-called crystal packing forces.

The naturally occurring hybrid promoter P*cg0441* has four consecutive thymines in the spacer region. This TTTT sequence should allow a significant bending of the spacer duplex since the AT base pairs are bound together by only two hydrogen bonds (in contrast to GC base pairs bound with three hydrogen bonds). Gaballa et al. identified a similar −35 proximal homo-polymeric T-tract as a novel promoter element involved in promoter discrimination by ECF σ factors in *B. subtilis*, likely by altering the trajectory of promoter DNA during engagement with RNAP [32]. Based on its conservation in a significant subset of deduced consensus sequences for ECF σs factors, they surmised that this element likely plays a general role in modulating the extent and impact of overlapping promoter recognition among ECF σ factors. Similarly, extended −10 promoters, which contain a 5’-TG-3’ element, tend to have longer spacers containing short runs of T residues. These promoters also show fewer matches to the consensus −35 hexamer element [33].

Considering both structural factors mentioned above (i.e. atypical C_−35_ and the TTTT sequence in the spacer region), we believe that they could jointly explain this rather unexpected and for us undesired recognition of the −10 region of P*cg0441* by σ^D^ (which undoubtedly has biological significance). Since, at this moment, we don’t see any simple way to modify the P*cg0441* sequence so that it was not recognized by σ^D^ but still was recognized by σ^H^_6aa_K, we tried a different approach to gain a hybrid promotor capable of bio-orthogonal transcription.

In subsequent experiments, we focused on two promoters, i.e. both σ^D^-dependent (P*rsdA*) and σ^H^-dependent (P*uvrD3*) that carry conventional −35 consensus sequences with G_−35_ in the first position (**G**GAAT and **G**TAAC). This G_−35_ is efficiently recognized by R175 in both σ^D^ and σ^H^ using two mutual hydrogen bonds that remarkably stabilize the positioning of the −35 element relative to σ subunits. As expected, σ^D^ stimulated transcription of σ^D^-dependent *PrsdA* by far the most strongly (transcription stimulated by σ^H^ or by its mutants was negligible). On the other hand, the σ^H^-dependent P*uvrD3* was not recognized by σ^D^ at all. Therefore, we created an artificial hybrid promoter combining the −10 element from the P*uvrD3* promoter and the −35 element from the P*rsdA* promoter which showed almost optimal properties in terms of bio-orthogonal transcription.

For the future, we see some space for potential further improvements in two ways. First, a model of the complete RNAP with the entire σ^H^_6aa_K and in complex with the P*D*_*35*_*H*_*10*_ hybrid promoter could allow the design of base mutations to improve the interaction between the P*D*_*35*_*H*_*10*_ hybrid promoter spacer and RNAP. Second, it could be interesting to make additional point mutations of σ^H^_6aa_K so that it is not recognized by the anti-sigma factor RshA [34, 35]. Our earlier results show that this could yield a several-fold increase in transcription [36]. However, both would require MD simulations of significantly larger molecular systems and detailed calculations of binding free energy [37, 38].

## Supplementary Information

Below is the link to the electronic supplementary material.

Supplementary file 1 (DOCX 101 kb)

Supplementary file 2 (DOCX 15 kb)

## Declarations

### Data Availability

No datasets were generated or analysed during the current study.
