# Supplementary material for: Promoter recognition specificity of Corynebacterium glutamicum stress response sigma factors σD and σH deciphered using computer modeling and point mutagenesis
Source: J Comput Aided Mol Des. 2024 Nov 25;39(1):1. doi: 10.1007/s10822-024-00577-x (PMC11588781; doi:10.1007/s10822-024-00577-x)
Supplement: Supplementary file 1 — (DOCX 101 kb) [file 10822_2024_577_MOESM1_ESM.docx]

16 nt spacer (n=3)


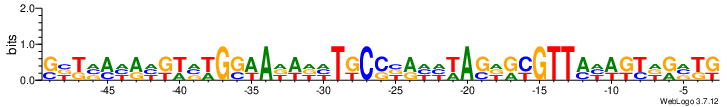

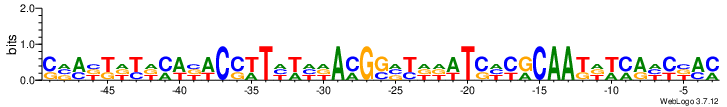


17 nt spacer (n=30)


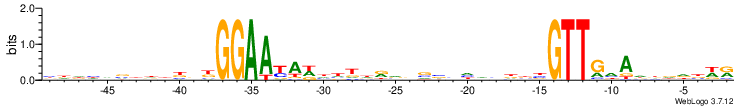

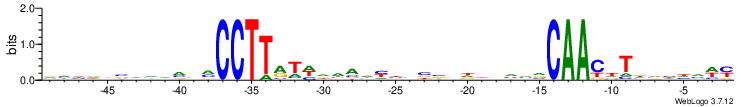


18 nt spacer (n=10)


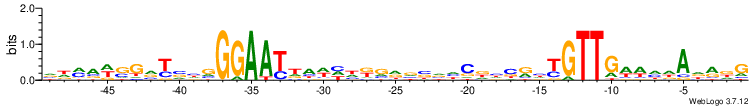


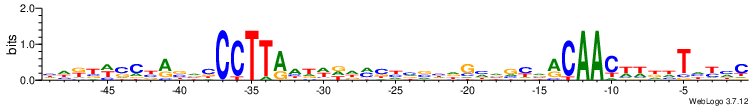


19 nt spacer (n=1)


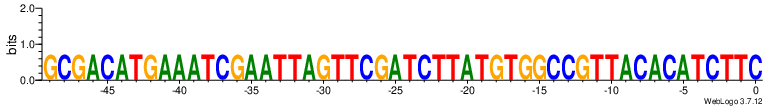

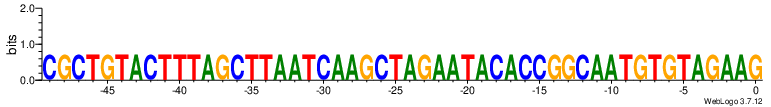


**Figure S1:** Regarding σ^H^-dependent promoters, the GGAAT/N_17-18_/GTT sequence is nearly completely conserved in the -35/spacer/-10 sequence.
