# Supplementary material for: Promoter recognition specificity of Corynebacterium glutamicum stress response sigma factors σD and σH deciphered using computer modeling and point mutagenesis
Source: J Comput Aided Mol Des. 2024 Nov 25;39(1):1. doi: 10.1007/s10822-024-00577-x (PMC11588781; doi:10.1007/s10822-024-00577-x)
Supplement: Supplementary file 2 — (DOCX 15 kb) [file 10822_2024_577_MOESM2_ESM.docx]

**Supplementary Table 1S:** List of oligonucleotides used in PCR mutagenesis

| **Oligonucleotide primer** | **Nucleotide sequence^a)^** |
| --- | --- |
| SIGHmut4AAF | **cgtcgca**CTCCATCGTGGAAGAAAACAG |
| SIGHmut4AAR | **cg**cac**agc**TCCGAGTGGAACGTCCAT |
| SIGHmut6AAF | gtg**cgcgtcgca**CTCCATCGTGGAAGAAAAC |
| SIGHmut6AAR | **agc**tcc**tggggt**AACGTCCATGATCTCGGC |
| SIGHmutE140KF | ACTCAGCCCG**AAG**TACCGGATGG |
| SIGHmutE140KR | TGATTCATTGCATCGCCAATTTTTC |

^a)^ Triplets or reverse complement triplets corresponding to altered amino acid residues are shown in bold.
